# Antibacterial Activity of Protocatechuic Acid Ethyl Ester on *Staphylococcus aureus* Clinical Strains Alone and in Combination with Antistaphylococcal Drugs

**DOI:** 10.3390/molecules200813536

**Published:** 2015-07-23

**Authors:** Maria Miklasińska, Małgorzata Kępa, Robert D. Wojtyczka, Danuta Idzik, Anna Zdebik, Kamila Orlewska, Tomasz J. Wąsik

**Affiliations:** Department of Microbiology and Virology, School of Pharmacy and Division of Laboratory Medicine in Sosnowiec, Medical University of Silesia, ul. Jagiellońska 4, 41-200 Sosnowiec, Poland; E-Mails: maria.miklasinska@o2.pl (M.M.); mkepa@sum.edu.pl (M.K.); rwojtyczka@sum.edu.pl (R.D.W.); didzik@sum.edu.pl (D.I.), anna.zdebik@gmail.com (A.Z.); kamila_orlewska@o2.pl (K.O.)

**Keywords:** *Staphylococcus aureus*, protocatechuic acid ethyl ester, antibacterial activity, synergistic effect

## Abstract

The aim of the presented study was to examine *in vitro* the antibacterial activity of protocatechuic acid ethyl ester (ethyl 3,4-dihydroxybenzoate, EDHB) against *Staphylococcus aureus* clinical isolates alone and in the combination with four selected antibiotics. The EDHB antimicrobial activity was tested against twenty *S. aureus* strains isolated from the clinical samples, and three reference strains. The phenotypes and genotypes of resistance to methicillin for the tested strains were defined as well as the phenotypic resistance to macrolides, lincosamides and streptogramin B (MLS_B_). EDHB displayed diverse activity against examined *S. aureus* strains with the minimal inhibitory concentration (MIC) within the range from 64 to 1024 µg/mL. Addition of ¼ MIC of EDHB into the Mueller-Hinton Agar (MHA) resulted in augmented antibacterial effect in the presence of clindamycin. In the case of cefoxitin no synergistic effect with EDHB was noted. For erythromycin and vancomycin the decrease of mean MICs in the presence of EDHB was observed but did not reach statistical significance. The results of the present study showed that *in vitro* EDHB possesses antibacterial activity against *S. aureus* clinical strains and triggers a synergistic antimicrobial effect with clindamycin and to the lesser extent with erythromycin and vancomycin.

## 1. Introduction

In the European Union nosocomial infections affect approximately 3 million people each year, and about 50,000 cases turn out to be fatal (data of European Centre for Disease Prevention and Control). Clinical environment modifies disease progression which results in the prolonged hospitalization (for 5–10 days) and increases the cost of treatments from 30% up to even 100% [1,2,3,4,5,6].

Staphylococci commonly colonize human body which makes them one of the major pathogens responsible for nosocomial infections [7]. Colonization significantly increases the risk of infection because colonized area forms a stable bacterial reservoir from which pathogens may spread posing danger, particularly to the immunocompromised patients [8]. Multi-drug resistant staphylococci are one of the major public health problems since the pathogens easily circulate in the environment [9]. Many errors in the implementation of anti-staphylococcal antibiotics and in the treatment strategies resulted in the selection and spread of drug resistant strains. Methicillin resistant *Staphylococcus* *aureus* (MRSA) strains pose a serious treatment problems among hospitalized patients. Moreover, bacteria resistant to glycopeptides antibiotics, which are the drugs of the last resort against MRSA, have been identified. An increased resistance to macrolide, lincosamide and streptogramin B (MLS_B_) antibiotics among staphylococci is also observed, what is a consequence of their extensive use in infections caused by Gram-positive bacteria [7]. Therefore, there is a strong necessity to find an alternative to the standard antimicrobial therapies. Many studies indicated that natural compounds in the combination with commonly used antibacterial drugs may constitute a new strategy in the treatment of infections caused by the multi-drug resistance bacteria. It has been proved that phytochemical compounds such as flavonoids or phenolic acids demonstrate antimicrobial properties against Gram-positive and Gram-negative bacteria [9,10,11,12,13,14,15,16,17,18,19].

EDHB is a polyphenol found in many plants, e.g., peanut seed testa. EDHB has been the subject of interest of the scientists for its various properties, including antibacterial and antioxidant [20], cardioprotective [21], myoprotective [22] anti-osteoporosis activity [23], chelating iron bounding enzymes [24] and modifying hypoxia tolerance [25]. Moreover, there are reports indicating that collagen production can be reduced by EDHB, due to its abilities to inhibit prolyl hydroxylase [26,27]. Breast cancer mouse model studies showed that EDHB can decrease metastasis and fibrosis acting as a proxyl hydroxylase inhibitor [28]. Recently, the reduction of the proliferation of esophageal squamous cell carcinoma in the presence of EDHB has been reported [29]. In addition, Muley *et al.* showed on the cerebral ischemic rat model that EDHB demonstrated neuroprotective properties by inhibiting radical damage to the neurons [30].

The antimicrobial activity of EDHB against *S. aureus*, in the contrast to that of protocatechuic acid (PCA), has not been definitely determined yet. Taking the above into consideration we have decided to evaluate *in vitro* antibacterial activity of EDHB alone and in combination with erythromycin (E), clindamycin (DA), cefoxitin (FOX) and vancomycin (VA) against 20 *S. aureus* clinical isolates and three reference strains: *S. aureus* ATCC 25923, *S. aureus* ATCC 43300 and *S. aureus* ATCC 6538.

## 2. Results and Discussion

All the examined strains were classified as members of *Staphylococcus aureus* species by classic microbiological methods and by Polymerase Chain Reaction-Restriction Fragment Length Polymorphism (PCR-RFLP) technique. For all the tested strains the phenotypic and genotypic resistance profiles to methicillin were assessed as well as the resistance phenotypes to macrolides, lincosamides and streptogramin B (Table 1). The majority of the analyzed strains were resistant to methicillin, presented *mecA* gene, and demonstrated the constitutive mechanism of resistance to MLS_B_ antibiotics.

### 2.1. Antibacterial Activity of the Protocatechuic Acid Ethyl Ester

In the presence of EDHB we observed the growth inhibition of all the tested staphylococci. The MIC values for EDHB ranged from 64 to 1024 µg/mL with a median of 512 µg/mL, lower quartile (LQ) 256 µg/mL and upper quartile (UQ) 1024 µg/mL (Table 2). *S. aureus* strains 7 and 2 showed the lowest MIC values: 64 µg/mL and 128 µg/mL, respectively. Five of 23 tested strains demonstrated MICs at 256 µg/mL. The most frequent MIC value among responding staphylococci was 512 µg/mL, and was demonstrated by 13 strains. The highest MICs (1024 µg/mL) were noted for *S. aureus* strains 10, 18 and 20. The MIC values against MSSA (methicillin susceptible *Staphylococcus aureus*) and MRSA strains ranged from 128 to 1024 µg/mL and from 64 to 1024 µg/mL respectively. The statistical analysis indicated that differences between MICs obtained for MRSA *vs.* MSSA strains were not significant (*p* = 0.531). Similarly, there were no significant differences between MICs values for MLS_B_ negative *vs.* kMLS_B_ and iMLS_B_ strains (*p* = 0.735).

### 2.2. Effect of Protocatechuic Acid Ethyl Ester in Association with Antibiotics against Staphylococcus aureus Strains

Combined *in vitro* effects of EDHB and erythromycin (E), clindamycin (DA), cefoxitin (FOX) and vancomycin (VA) are shown in Table 3. The statistical analysis showed that the addition of one-fourth of the MIC of EDHB to the MHA medium significantly increased sensitivity of the examined strains to DA (*p* = 0.038). The decrease of MICs after EDHB addition to the MHA was noted also for E (*p* = 0.306) and VA (*p* = 0.196), but these results did not reach the statistical significance, while for FOX and EDHB the opposite trend was observed (*p* = 0.328).

The most noticeable decreases of MICs were observed for six *S. aureus* strains (1, 4, 5, 7, 9, and 10). *S. aureus* strain 4 was found to be more sensitive to all antibiotics after EDHB addition with the decrease of MICs ranging from 25% (for VA) to 50% (for E, DA and FOX). In the case of the *S. aureus* strain 5, the decrease of MICs after MHA supplementation with E and DA was observed (94% and 83% decrease, respectively), while MICs of VA and FOX were not changed.

**Table 1:** *Staphylococcus aureus* susceptibility to methicillin and MLS_B_ antibiotics assessed by the disc diffusion method (Cefoxitin test and D-test) and molecular technique (detection of *mecA* gene).

Strain	Cefoxitin Diameter of the Inhibition Zone [mm]	Presence of *mecA*	Methicillin Resistance Profile	Erythromycin Diameter of the Inhibition Zone [mm]	Clindamycin Diameter of the Inhibition Zone [mm]	Mechanism of Resistance to MLS_B_ Antibiotics
*S. aureus* ATCC 25923	35	−	MSSA	25	25	-
*S. aureus* ATCC 43300	21	+	MRSA	0	0	kMLS_B_
*S. aureus* ATCC 6538	31	+	MRSA	30	30	-
*S. aureus* 1	34	−	MSSA	25	25	-
*S. aureus* 2	32	−	MSSA	23	25	-
*S. aureus* 3	31	−	MSSA	0	25	iMLS_B_
*S. aureus* 4	32	+	MRSA	25	27	-
*S. aureus* 5	13	+	MRSA	0	30	iMLS_B_
*S. aureus* 6	31	−	MSSA	30	35	-
*S. aureus* 7	32	+	MRSA	35	33	-
*S. aureus* 8	31	−	MSSA	30	35	-
*S. aureus* 9	30	+	MRSA	35	25	-
*S. aureus* 10	31	−	MSSA	10	22	iMLS_B_
*S. aureus* 11	31	−	MSSA	21	22	-
*S. aureus* 12	8	+	MRSA	0	0	kMLS_B_
*S. aureus* 13	14	+	MRSA	0	0	kMLS_B_
*S. aureus* 14	0	+	MRSA	0	0	kMLS_B_
*S. aureus* 15	21	+	MRSA	25	30	-
*S. aureus* 16	18	+	MRSA	0	0	kMLS_B_
*S. aureus* 17	11	+	MRSA	0	0	kMLS_B_
*S. aureus* 18	19	+	MRSA	25	30	-
*S. aureus* 19	14	+	MRSA	0	0	kMLS_B_
*S. aureus* 20	19	+	MRSA	0	0	kMLS_B_

MRSA: methicillin resistance *Staphylococcus aureus*, MSSA: methicillin susceptible *Staphylococcus aureus*, kMLS_B_: constitutive macrolide, lincosamide and streptogramin B mechanism, iMLS_B_: inducible macrolide, lincosamide and streptogramin B mechanism.

**Table 2:** MIC (µg/mL) of protocatechuic acid ethyl ester against *S. aureus* strains.

Bacterial Strain	EDHB MIC (µg/mL)
*S. aureus* ATCC 25923	256
*S. aureus* ATCC 43300	512
*S. aureus* ATCC 6538	256
*S. aureus* 1	512
*S. aureus* 2	128
*S. aureus* 3	256
*S. aureus* 4	512
*S. aureus* 5	512
*S. aureus* 6	512
*S. aureus* 7	64
*S. aureus* 8	512
*S. aureus* 9	512
*S. aureus* 10	1024
*S. aureus* 11	512
*S. aureus* 12	256
*S. aureus* 13	512
*S. aureus* 14	512
*S. aureus* 15	512
*S. aureus* 16	512
*S. aureus* 17	256
*S. aureus* 18	1024
*S. aureus* 19	512
*S. aureus* 20	1024
Median	512
LQ–UQ	256–1024

EDHB: ethyl 3,4-dihydroxybenzoate, MIC: minimal inhibitory concentration, LQ: lowest quartile, UQ: upper quartile.

The growth of *S. aureus* strain 1 was from 25% to 37% (excluding E) after addition of EDHB to MHA medium. Analyzing the susceptibility of *S. aureus* strain 10 we noted for DA, FOX, and VA substantial reduction of MICs from 13% to 66%, while MIC for E was not changed. The effect of the interaction of EDHB and antibiotics on *S. aureus* strain 7 resulted in the decrease of MICs by 34% for E, 50% for DA and 25% for FOX. The augmented effect of EDHB was also observed for *S. aureus* strain 9 in the presence of E (MIC decrease of 50%), DA (MIC decrease of 50%) and FOX (MIC decrease of 13%). 

For some staphylococci strains and some antibiotics in the presence of EDHB the increase of MICs was noted. *S. aureus* ATCC 25923 strain in the presence of EDHB showed lower sensitivity to E with the MIC increase by 32%, to DA by 47%, and to VA by 25%. The similar increase was noted for *S. aureus* ATCC 43300 strain for FOX by 167%, and for VA by 37% and in case of *S. aureus* strain 15 for E by 100%, and for FOX by 50%. The level of resistance to FOX, E and DA was not affected by the presence of EDHB for seven *S. aureus* strains (12, 13, 14, 16, 17, 19, 20) (Table 3).

**Table 3:** *Staphylococcus aureus* susceptibility to methicillin and MLS_B_ antibiotics assessed by disc diffusion method (Cefoxitin test and D-test) and molecular technique (detection of *mecA* gene).

Bacterial Strain	E	E + EDHB	∆%	DA	DA + EDHB	∆%	FOX	FOX + EDHB	∆%	VA	VA + EDHB	∆%
*S. aureus* ATCC 25923	0.38	0.50	−32	0.064	0.094	−47	1	1	0	0.75	1	−25
*S. aureus* ATCC 43300	256	256	0	256	256	0	12	32	−167	0.38	0.75	−37
*S. aureus* ATCC 6538	0.064	0.094	−47	0.023	0.023	0	2	1.5	25	0.50	0.38	12
*S. aureus* 1	0.50	0.50	0	0.064	0.047	27	2	1.5	25	0.75	0.38	37
*S. aureus* 2	0.50	0.38	24	0.064	0.047	27	0.75	1.5	−100	0.38	0.38	0
*S. aureus* 3	256	256	0	0.023	0.047	−104	1.5	1.5	0	0.50	0.38	12
*S. aureus* 4	0.38	0.19	50	0.064	0.032	50	2	1	50	0.50	0.25	25
*S. aureus* 5	256	16	94	0.094	0.016	83	256	256	0	0.75	0.75	0
*S. aureus* 6	0.50	0.38	24	0.064	0.064	0	1.5	2	−33	0.38	0.50	−12
*S. aureus* 7	0.38	0.25	34	0.032	0.016	50	1	0.75	25	0.50	0.50	0
*S. aureus* 8	0.19	0.38	−100	0,032	0,016	50	1.5	0.75	50	0.38	0.38	0
*S. aureus* 9	0.38	0.19	50	0.064	0.032	50	1	1	0	0.38	0.25	13
*S. aureus* 10	32	32	0	0.047	0.016	66	2	1	50	0.38	0.25	13
*S. aureus* 11	0.38	0.19	50	0.047	0.047	0	1.5	2	−33	0.38	0.19	19
*S. aureus* 12	256	256	0	256	256	0	256	256	0	0.75	0.50	25
*S. aureus* 13	256	256	0	256	256	0	32	256	−700	0.75	0.50	25
*S. aureus* 14	256	256	0	256	256	0	256	256	0	0.75	0.50	25
*S. aureus* 15	0.25	0.50	−100	0.064	0.032	50	8	12	−50	0.38	0.38	0
*S. aureus* 16	256	256	0	256	256	0	256	256	0	0.50	0.75	−25
*S. aureus* 17	256	256	0	256	256	0	256	256	0	0.38	0.38	0
*S. aureus* 18	0.38	0.38	0	0.047	0.023	51	6	6	0	0.50	0.50	0
*S. aureus* 19	256	256	0	256	256	0	256	256	0	0.50	0.38	12
*S. aureus* 20	256	256	0	256	256	0	12	256	−2033	0.38	0.50	−12
median	0.5	0.5	0	0.064	0.047	0	2	2	0	0.50	0.38	0
LQ	0.38	0.38	0	0.047	0.023	0	1.5	1.25	−33	0.38	0.38	0
UQ	256	256	94	256	256	84	256	256	50	0.75	1	37
*p*	0.306			0.038			0.328			0.196		

EDHB: ethyl 3,4-dihydroxybenzoate, E: erythromycin, DA: clindamycin, FOX: cefoxitin, VA: vancomycin, LQ: lowest quartile, UQ: upper quartile.

Statistical analysis revealed no significant differences between MICs changes for MRSA *vs.* MSSA strains (E − *p* = 0.862; DA − *p* = 0.606; FOX − *p* = 0.566; VA − *p* = 1.000), as well as for MLS_B_ negative *vs.* kMLS_B_ and iMLS_B_ strains (E − *p* = 0.597; DA − *p* = 0.083; FOX − *p* = 0.154; VA − *p* = 0.939) (Table 3). The presented study on the association of the antibacterial action of EDHB with the selected antibiotics showed that the most significant synergistic effect was noted between EDHB and DA. The synergism between EDHB and antibiotics was noted also for E and VA, but it did not prove to be statistically significant. The combined effect between EDHB and FOX was not observed.

### 2.3. Discussion

The spread of the drug resistance among *S. aureus* strains has led to the intensified search for the new antibacterial agents. Many studies on the synergism between the diverse substances produced by a living organism and the common antibiotics showed that *S. aureus* strains were sensitive to some of them to the varying degrees [11,12,13,14,15,16,17,18,19].

The majority of the research focused on the antimicrobial properties of protocatechuic acid (PCA), while EDHB has not been widely studied yet. Many authors have proved PCA antibacterial activity against both Gram-positive and Gram-negative bacteria [31,32,33,34,35,36,37]. In this case, our work seems to be one of the first studies which concentrate on the antibacterial effect of EDHB alone, and in combination with the selected antibiotics.

The antibacterial activity of PCA has been proven against *E. coli*, *P. aeruginosa*, *S. aureus*, *B. cereus*, *K. pneumoniae*, *A. baumannii* [31,32,36]. Jayaraman *et al.* showed that MICs of PCA were lower than MICs of rutin or berberin against *P. aeruginosa* strains. Moreover, they noted that PCA and sulfamethoxasole exhibited synergistic effect against *P. aeruginosa* strains [32]. 

The study carried out by Chao *et al.* showed that PCA inhibited development of *S.* Typhimurium, *B. cereus*, *L. monocytogenes*, *S. aureus* and *E. coli* when added to the ground beef and apple juice. The authors proposed that PCA might be a new potent antimicrobial agent added to the food to eliminate the contamination by these pathogens [34]. It has also been reported that PCA showed *in vitro* activity against a range of bacteria such as MRSA, *K. pneumoniae*, *P. aeruginosa*, *A. baumannii* causing nosocomial infections [35] and *H. pylori* [36,37]. 

The study on antimicrobial activity of EDHB was carried out by Merkl *et al.* [20].The authors compared antibacterial effect of the phenolic acids and their alkyl esters, against *E. coli*, *B. cereus*, *L. monocytogenes*, *S. cerevisiae* and *F. culmorum* and reported significant differences between MICs of PCA and its esters. EDHB proved to be two times more active against *E. coli* (MIC-5 mmol/L) than PCA (MIC-10 mmol/L) and *F. culmorum* and *S. cerevisiae* were eight times more susceptible to EDHB than to PCA, with MIC values at 2.5 mmol/L and ≥20 mmol/L, respectively. In the case of *B. cereus* and *L. monocytogenes*, there were no differences in MICs between acid and esters, 5 mmol/L in both cases. Moreover, the authors reported that the increase of the antibacterial activity was associated with the increase of ester’s alkyl chain length [20]. 

The present study has demonstrated that EDHB possesses antibacterial activity against clinical *S. aureus* strains. The MICs of EDHB against *S. aureus* strains ranged from 64 to 1024 µg/mL. The statistical analysis excluded the possibility that the dispersion of the results was affected by methicillin resistance profile or phenotype of resistance to MLS_B_ antibiotics. It is probable that the varying sensitivity of the examined strains to EDHB was due to the ontogenetic diversity within the species. Because all MRSA strains carried *mecA* gene it can be assumed that the presence of this gene did not influence EDHB action. One may assume that the large discrepancies of MIC values were caused by the type of SCC*mec* cassette or the presence of other resistance genes. However, since EDHB mechanism of action on the bacterial cell is still unknown, we can only speculate what may be responsible for the observed interactions. The future studies are needed to determine EDHB mechanism of action against the bacterial cell and its potential application in the therapies for *S. aureus* infections.

Our data proved significant synergistic effects between EDHB and DA. The synergism between EDHB and erythromycin and vancomycin was also noted, but it did not reach statistical significance. We think that antagonistic trend observed for EDHB and FOX might have been caused by competitive inhibition, since it is possible that FOX and EDHB have the same binding site in bacterial cells. 

It is believed that natural compounds with higher MICs than antibiotics cannot be used in antibacterial monotherapy for their insufficient therapeutic effect. Nevertheless, their use in the combination with antibiotics can significantly decrease the spread of drug resistant bacterial strains by modulating the resistance mechanisms and augmenting the effectiveness of antimicrobial therapy. Implementation of the combined therapy can increase the potential of antibiotics by the improvement of their pharmacokinetic and pharmacodynamic properties. Moreover, it may contribute to the reduction of the drug dosage, thus diminishing side effects of antibiotics. The results of the present study imply that it is important to undertake the future research to understand EDHB antimicrobial mechanism of action and to determine its full potential for the *S. aureus* infections treatment.

## 3. Experimental Section

### 3.1. Bacterial Strains and Protocatechuic Acid Ethyl Ester

The antimicrobial activity of EDHB was assessed against 20 *S. aureus* strains isolated from clinical wound samples, and three reference strains of *S. aureus*: ATCC 25923, *S. aureus* ATCC 43300 and *S. aureus* ATCC 6538. To ensure the homogeneity of the analyzed samples all the examined strains were collected from surgical wounds. All the tested strains were stored in the Trypticase Soy Broth medium with 20% of glycerol at −80 °C. EDHB used in this study was received from Sigma Chemical Co. (St. Louis, MO, USA) and dissolved in DMSO immediately prior to use.

### 3.2. Identification of Examined Strains

The examined strains were identified by the standard microbiological methods such as hemolysis, catalase and coagulase tests and the anaerobic fermentation of mannitol, then the API STAPH test was used (bioMerieux, Marcy-l’Étoile, France) according to the manufacturer’s instruction. To ensure that the clinical strains were identified unambiguously the PCR-RFLP method was performed. GeneMATRIX Tissue & Bacterial DNA Purification KIT (EuRx Ltd., Gdańsk, Poland) was used for the bacterial genomic DNA isolation according to the manufacturer’s recommendations with the modification described by Shah *et al.* [38]. To amplify the *dnaJ* gene fragment the primers SA-(F) 5′-GCC AAA AGA GAC TATTAT GA-3′ and SA-(R) 5′-ATT GTT TAC CTG TTT GTG TAC C-3′ were used. PCR reaction was performed using 10× PCR RED master mix kit (BLIRT SA, Gdańsk, Poland) in a MJ Mini Personal Thermal Cycler (Bio-Rad, Hercules, CA, USA). To separate PCR products, the electrophoretic separation in a 1.5% agarose gel (Promega, Madison, WI, USA) with ethidium bromide (Promega) was performed, PCR products were visualized under the UV light. To confirm the classification of species, the specific restriction profiles after cleaving of PCR products with 10U of restriction enzymes *Xap*I and *Bsp*143I (Fermentas, Vilnius, Lithuania) were analyzed. Briefly, the restriction fragments were separated by the electrophoresis in a 2% agarose gel with ethidium bromide (Promega). Their size was checked against 1 Kb HypeLadderIV (BLIRT SA) molecular weight marker and visualized under the UV light.

### 3.3. Phenotypic and Genotypic Resistance to Methicillin

The resistance phenotypes to methicillin were determined according to the disc diffusion method with cefoxitin 30 µg disc (EMAPOL, Gdańsk, Poland) and Mueller-Hinton Agar (MHA-BTL, Łódź, Poland). All strains were classified as MRSA or MSSA according to the zone diameter size (≤22 and >22, respectively). The PCR detection of the *mecA* gene was carried out according to the method described by Murakami *et al.* with the use of the following primers (F) (5′-AAA ATC GAT GGT AAA GGT TGG C-3′) and (R) (5′-AGT TCT GCA GTA CCG GAT TTG C-3′) [39]. PCR was performed using 10× PCR RED master mix kit (BLIRT SA) in a MJ Mini Personal Thermal Cycler (Bio-Rad). The identification of amplicons was carried out by the electrophoresis in 1.5% agarose gel with ethidium bromide (Promega) and visualized under UV light.

### 3.4. Antimicrobial Susceptibility Testing 

The antimicrobial susceptibility of *S. aureus* strains to macrolides and lincosamides was tested by disk-diffusion method and interpreted according EUCAST guidelines [40]. The commercial antibiotic discs (EMAPOL) and Mueller-Hinton Agar (MHA-BTL) were used in this tests. A colony suspension equivalent to 0.5 McFarland unit was inoculated to Mueller-Hinton Agar with a 15 μg clindamycin (DA) and 2 μg erythromycin (E) disks. According to EUCAST recommendation distance between the edges of disks was 15–16 mm. The zone diameter size was interpreted after 18 h of incubation at 35 °C. The strains were classified as resistant or sensitive based on the zone diameter size and shape.

### 3.5. Microdilution Method

The MICs of EDHB were measured by the broth microdilution liquid method. The growth inhibition assays were performed in sterile 96-well plates (FL Medical, Torreglia, Italy) in the final volume of 200 µL [41,42]. The cell concentrations were assessed from the optical densities at 600 nm wavelength with the formula CFU/mL = *A*_600_ (3.8 × 10^8^), where CFU was the number of colony-forming units. One hundred microliters of mid-logarithmic-phase bacterial cultures (5 × 10^5^ CFU/mL) in TSB was added to 100 μL of sterile serially diluted EDHB (1, 2, 4, 8, 16, 32, 64, 128, 256, and 1024 μg/mL). Wells containing TSB with bacterial inoculum only served as a bacterial growth control (GC). The additional control included TSB alone, as a medium sterility control, and TSB with different concentrations of EDHB and bacterial inoculum. All the samples were prepared and measured in triplicates. Microplates were incubated at 37 °C for 24 h, and the bacterial cell growth was assessed by measuring the optical density of cultures at 600 nm wavelength with a Multiskan EX microplate reader (Thermo Electron Corp., Vantoa, Finland) [43,44]. MICs were defined as the lowest EDHB concentration that completely inhibits bacterial growth [41,42].

### 3.6. Combined Effect of EDHB and Antibiotics on S. aureus Strains

All the strains were tested for the antimicrobial susceptibility by the E-test method, using MHA and commercially available MIC Test Strips (Liofilchem, Roseto Degli Abruzzi, Italy) containing antibiotic concentration gradient according to the EUCAST recommendations [40]. For E-test method, 90 mm plates with the agar medium were inoculated by swabbing the agar with a swab soaked in a bacterial suspension of 1× 10^8^ cells/mL. MIC Test Strips containing concentration gradient of erythromycin (E), clindamycin (DA), cefoxitin (FOX) and vancomycin (VA) were used for the analysis of *S*. *aureus* antimicrobial susceptibility.

The combined effect of EDHB and antibiotics was studied using plates with MHA plus one-fourth of the MIC of EDHB, which was considered as a sub-inhibitory concentration [45,46]. The test strips were placed onto an agar surface and gently pressed using sterile forceps to ensure the contact. Plates were incubated at 35 °C for 20 h in the aerobic condition. The susceptibility testing of each antibiotics for each isolates and the reference strains was performed in triplicates. After the incubation MIC values were read and the median values were calculated.

### 3.7. Statistical Analysis

To compare MICs and MICs changes across MRSA and MSSA U Mann-Whitney test was used and the Kruskal-Wallis tests was used to compare MICs and MICs changes across strains negative for MLS_B_, kMLS_B_ and iMLS_B_. The results from the synergism assay were submitted to the Wilcoxon Signed-Rank Test. For all used test *p* ≤ 0.05 was considered as statistically significant. The data was analyzed with the use of STATISTICA v 10.0 (StatSoft, Krakow, Poland).

## 4. Conclusions 

Our data showed that EDHB exerted antimicrobial activity towards clinical isolates of *S. aureus* and the observed effect is varied among the strains. The presence of EDHB significantly augmented clindamycin antimicrobial activity which strongly suggests that the synergistic effect between EDHB and DA toward *S. aureus* strains may enhance the action of this antibiotic *in vivo*. This promising observation implies that the further study focused on determining the precise mechanisms of antimicrobial activity of EDHB towards *S. aureus* strains should be performed. Such a research should contribute to the development of the new therapies effective against multidrug resistant *S. aureus* clinical strains.
